# Rheologically Assisted Design of Conductive Adhesives for Stencil Printing on PCB

**DOI:** 10.3390/ma14247734

**Published:** 2021-12-15

**Authors:** Ângelo D. M. Silva, Mariana M. Silva, Hugo Figueiredo, Isabel Delgado, Paulo E. Lopes, Maria C. Paiva, Loic Hilliou

**Affiliations:** 1 Instituto de Polímeros e Compósitos (IPC), Universidade do Minho, 4804-533 Guimarães, Portugal; a77129@alunos.uminho.pt (Â.D.M.S.); mmsilva@dep.uminho.pt (M.M.S.); mcpaiva@dep.uminho.pt (M.C.P.); 2 Bosch Car Multimedia Portugal SA (CM/MFT1), 4705-820 Braga, Portugal; Hugo.Figueiredo2@pt.bosch.com (H.F.); Isabel.Delgado@pt.bosch.com (I.D.)

**Keywords:** solder paste, electrically conductive adhesives, carbon nanotubes, exfoliated graphite, stencil printing, yield stress, printed circuit boards, rheology

## Abstract

Driven by the need to deliver new, lead-free, eco-friendly solder pastes for soldering electronic components to Printed Circuit Boards (PCB), electrically conductive adhesives (ECAs) based on epoxy, carbon nanotubes (CNT), and exfoliated graphite (EG) were designed. The rheology of the adhesives prepared is paramount for the success of the deposition process, which is based on stencil printing. Thus, a rheological analysis of the process was first performed. Then, an experimental protocol was defined to assess the relevant viscoelastic characteristics of the adhesives for stencil printing application. Different composite formulations of epoxy/CNT/EG were produced. Their rheological characteristics were established following the designed protocol and benchmarked with a commercial solder paste. The thermal and electrical properties of the composite formulations were also characterized. As a result, a new, electrically conductive adhesive was delivered with potential to be an eco-friendly alternative to the solder paste currently used in stencil printing of PCB.

## 1. Introduction

Surface mount technology (SMT) is an assembly process used for the placement and soldering of electronic components and devices on printed circuit boards (PCB). SMT allows the high-yield manufacture of, e.g., mobile phones and laptop computers and is the dominant electronics manufacturing assembly method. SMT is essentially a three-step process [1]. First, solder paste patterns are stencil printed on the PCB. Then, electronic components are placed over the patterns, and the whole assembly runs through a reflow soldering oven to melt the solder paste and eventually solder the components to the PCB pads.

Limitations such as the soldering of thermally sensitive components and voiding in the solder triggered the need for designing a hybrid SMT process where both solder pastes and electrically conductive adhesives (ECA) are used. Recently, ECA formulated with carbon nanotubes and graphite nanoplatelets (GNP) have been developed in view to replace the metallic fillers currently used in ECA designed for hybrid SMT [2]. GNP were shown to be essentially responsible for improved thermal conduction, whereas carbon nanotubes contributed more to the electrical conductivity. Here, we aimed at optimizing ECA formulations based on single-wall carbon nanotubes (SWCNT) and GNP in view to meet the SMT requirements. SWCNT were preferred to multi-wall carbon nanotubes since larger electrical conductivities were found for ECA formulated with SWCNT over a similar range of carbon nanotube content [2]. The optimization encompasses a fine-tuning of the viscoelastic properties of ECAs to the printing and placements steps. This can only be achieved after a rheological analysis of these two processes to define the pertinent viscoelastic functions to be tuned and the values of rheological parameters to be used during testing.

The rheology of stencil or screen printing has been the subject of many studies (see, e.g., [3,4] and references therein). Stencil printing is usually divided into two consecutive steps. First, a squeegee is pressed on the stencil and travels with a controlled velocity to spread the solder paste. During this step, which shares similarities with the spreading of material during screen printing, a roll of flowing paste is formed in front of the squeegee, which injects the paste through the apertures of the stencil. Finally, the stencil separates from the substrate, thus transferring the injected paste as printed deposits on the substrate. Relationships between the rheology of pastes and the quality of the printing have been established [5,6,7], and a printability map for solder pastes was proposed [8]. The rheological modelling of the process was also earlier performed [9,10,11] and improved by taking on board the vast literature on the rheology of solder pastes (see, e.g., [12] and references therein). The latter are solid materials that flow in a shear thinning and thixotropic manner when submitted to a stress larger than the yield stress and recovers their elastic characteristics after flow cessation.

The review of the short-listed literature referenced above indicated that shear rates and pressure of the order of 1000 s^−1^ and 10^5^ Pa, respectively, develop in the thixotropic and shear thinning pastes close to the squeegee’s tip. However, shear rates and stresses at play during the filling of apertures, the stencil separation from the substrate, and the force-controlled placement of electronic components on paste pads are much less documented, as these are specific to PCB designs and electronics used in a dedicated SMT line. Indeed, the need for the tuning of solder paste formulation, and, thus, its rheology, to the aperture size range has been highlighted [13]. Similarly, typical processing times for printing strokes’ cycles, filling and emptying apertures, or time lapse between components’ placements on the paste pad and soldering step are less documented. Such times are essential for tuning the paste thixotropy, as characteristic times for recovering the elastic character of the paste after flowing should match those specific process times. Note that times of the order of tens of milliseconds were reported for the levelling of the line shapes of paste deposits [6].

The rheological characterization of solder paste usually encompasses step shear rate tests (including jumps and intermittent shear) [6,8], creep and recovery [6,14], ramps in steady shear [4,5,7,8,13,14] with thixotropic hysteresis loops [8,13,14], and small [4,5,13,14] and large amplitude oscillatory shear [4,5,13]. More recently, extensional testing [15] was reported, as extensional deformation is considered important during stencil lifting, though such rheometry is very demanding for pastes. Here, an alternative experimental approach was used. Process parameters were quantified for a specific surface mounting technology line and PCB designs operated and developed in the facilities of Bosch Car Multimedia Portugal S.A. A rheological analysis of stresses, shear rates, and characteristic times involved during the stencil printing and electronics placement steps was performed in view of designing the rheometry to be used for the rheological study of pastes. The rheological analysis of the SMT before reflow soldering and the delivery of the corresponding protocol for paste rheometry were the basis for designing various formulations of ECA, aiming at benchmarking the linear and nonlinear viscoelastic properties of a commercial solder paste. ECA formulations that best matched the commercial solder paste were then thermally and electronically characterized. As a result, a route for solving thermal incompatibility in reflow soldering was proposed, based on the use of a solder paste and a rheologically designed ECA.

## 2. Materials and Methods

### 2.1. Materials

The ECA matrix is made of a three components’ thermoset, consisting of Biresin CR141 (1.16 g cm^−3^ density, viscosity 8.2 Pa.s, both at 25 °C), Biresin CH141 (which is a hardener with a density of 1.20 g cm^−3^ and a viscosity of 40 mPa.s, both at 25 °C), and Biresin CA141 (an accelerator, viscosity of 200 mPa.s and density of 0.98 g cm^−3^ both at 25 °C). These three components were sourced from Sika (Bad Urach, Germany).

Graphite nanoplatelets of xGnP^®^ grade M25, with average thickness of approximately 8 nm and average particle diameter of 25 µm, were bought from XG Sciences, Inc., (Lansing, MI, USA). Single wall carbon nanotubes Tuball™ were supplied by OCSiAl (Leudelange, Luxembourg). The producer’s specifications reported a purity larger than 75%, a SWCNT length larger than 5 µm, and an outer mean diameter of 1.8 ± 0.4 nm

The commercial solder paste used was SAC305 from Heraeus (Hanau, Germany).

### 2.2. Preparation of ECA

Biresin CR141, Biresin CH141, and Biresin CA141 were mixed together at a weight ratio of 100:90:2. The pot life of this mixture is nearly 24 h at room temperature and shows a viscosity of 600 mPa∙s. The carbon fillers and the resin components were all manually mixed in a beaker. This composite was then fed to a three-roll mill EXAKT 80E (EXAKT Advanced Technologies GmbH, Norderstedt, Germany), and a six-step program described in previous work [2] was used to achieve a good dispersion of the carbon nanofillers in the epoxy matrix. After dispersion with the three-roll mill, the composites were degassed in a vacuum chamber during 15 min to get rid of air bubbles. A list with the compositions of the ECA prepared and identifications used is presented in Table 1. The content of SWCNT was here limited to 0.5 wt.% since adding larger amounts would lead to depressed electrical conductivities, whereas the synergism effect between GNP and SWCNT on the shear elastic modulus was lost, as shown in detail elsewhere [2].

The rheological characterization of commercial solder pastes and ECA was carried at 25 °C with a stress rheometer (ARG2, TA Instruments, New Castle, DE, USA). Though slip at the interface between the moving walls of the stencil and the solder paste was early recognized and qualitatively analyzed during aperture emptying [11], very few rheological studies addressed the slip issue during the testing of solder pastes and ECA [4,14]. Here, aluminum sand-blasted and disposable plates (40-mm diameter) were used as shearing accessories to limit wall slip. The use of a vane geometry [14] was discarded as non-laminar flow and very large torques at large rotation speeds impede proper rheological testing at larger shear rates. Many other experimental artefacts are known to pollute the rheological analysis of concentrated colloidal suspensions [16]. Here, for a rapid preliminary assessment of possible experimental flaws, tests were carried out with a sample thickness of 800 microns and repeated with another sample and a smaller gap between the shearing plates. For each material, a sample was loaded between the plates, and the gap was adjusted within 10 microns above the targeted sample thickness. Then, the sample was trimmed and the gap was set to the defined value. Time was then allowed for the relaxation of the normal forces before initiating measurements.

A KEITHLEY SMU 2635B (Keithley Instruments Inc., Cleveland, OH, USA) current/voltage source and meter was used to acquire I-V curves during the ramping of the voltage between −10 and +10 V at 0.5 V steps. ECA samples with S3A geometry (dog-bone shaped as defined in the DIN 53,504 standard) were produced by casting ECA on a silicon mold and cured in a ventilated oven at 150 °C for 60 min. The geometry of the ECA samples and electric contacts between ends of the dog-bone-shaped samples and electrodes were taken into account for the computation of samples’ resistivity.

Differential scanning calorimetry (DSC) curves were acquired with a Netzsch 200 F3 calorimeter (Netzsch-Gerätebau GmbH, Selb, Germany). The cure of ECA samples was studied using a protocol detailed at length elsewhere [2]. It was based on three different types of heating/cooling tests performed with N_2_ as purge gas to characterize the epoxy cure, to simulate the PCB reflow, and to study the isothermal cure at 150 °C.

## 3. Results and Discussion

### 3.1. Rheological Analysis of the Stencil Printing and Electronic Components’ Placement

**Figure 1:**
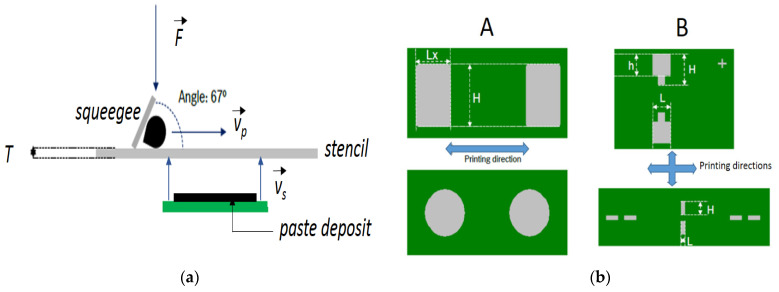
Parameters used for the stencil printing and separation from the PCBs (**a**) and schematic representation of the two designs A and B used for the stencil apertures (where Lx, H, h, and L identify the lengths and widths of various paste deposits x, in gray, on the same PCB, in green) (**b**).

The printing parameters and stencil designs used at the industrial plant for the stencil printing and component placements are portrayed in Figure 1. Note here that the shear and extension histories related with the semi-manual dispensing of material on the stencil before another cycle of prints were not considered, as they are difficult to quantify. Nonetheless, a rest time of *t_r_* = 2.5 s after dispensing was observed before starting stencil printing. Equally, the extensional rates associated with the separation of the stencil from the PCB were not considered. Two stencils with thickness *T* of 0.05 and 0.08 mm were used. The squeegee was submitted to a force F = 75 N and traveled at a velocity *v_p_* ranging from 25 to 50 mm.s^−1^, whereas the separation velocity vs. varied between 1 and 5 mm.s^−1^. The shear rates $\dot{\gamma_{p}}$ involved during printing were obtained through the relation $\dot{\gamma_{p}}=\frac{v_{p}}{T}$, whereas the shear rates $\dot{\gamma_{s}}$ involved during separation were obtained through the relation $\dot{\gamma_{s}}=\frac{v_{s}}{X}$, where *X* is the half-length, half-width or radius of the apertures. Given the footprints of the stencil design and the velocities, the ranges of shear rates applied on the solder paste or ECA were as follows: 312 s^−1^ < $\dot{\gamma_{p}}$ < 1000 s^−1^ and 0.16 s^−1^ < $\dot{\gamma_{s}}$ < 16 s^−1^.

A pick-and-place machine allowed the placement of electronic components on the printed solder paste and ECA deposits with a controlled force ranging from 2 to 15 N. Assuming for simplicity that this force was applied on the areas of the deposits and that the squeezing of components involved more shear than biaxial extension, shear stresses *σ_p_* ranging from 50 kPa to 62.5 MPa were applied during a time *t_p_*.

Three characteristic times of the SMT still needed to be defined as they condition the structural and rheological recovery of the printed materials under different conditions. The first was the time lapse *t_l_*_1_ between stencil printing and PCB separation. The second was the time *t_l_*_2_ needed to transfer the printed PCB from the printer to the pick-and-place machine. This time is usually of the order of a minute or more, as the quality of the printing is often controlled before placing the electronic components. The third was the duration *t_l_*_3_ of the conveying of the PCB from the pick-and-place machine to the reflow soldering oven. During *t_l_*_3_, the solder paste or ECA was loaded with the weight of the electronic component, which corresponded to a maximum stress of 206 Pa. However, the rotational rheometer used in the present study did not allow for measuring the flow or displacement of the sample under a compressive load of the order of 200 Pa. This was in contrast with the sample viscoelastic recovery during *t_l_*_1_ or *t_l_*_2_, which can be measured as detailed below.

### 3.2. Design of the Rheological Protocol

**Figure 2:**
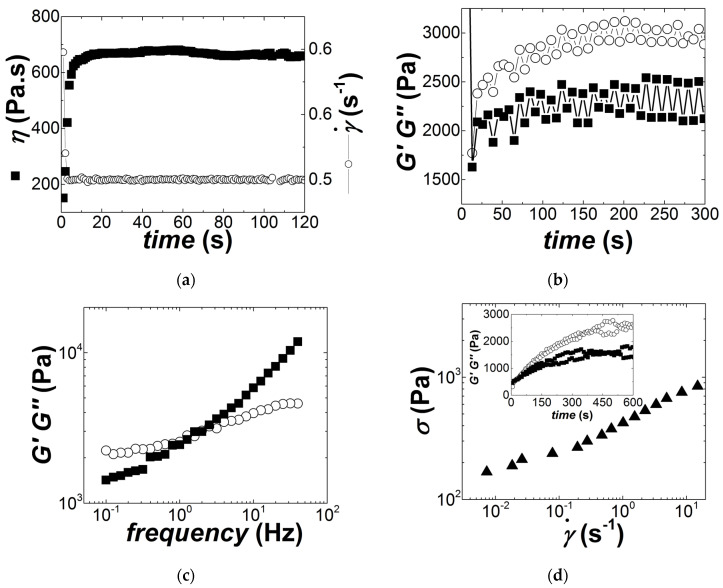
Rheological protocol carried out with a sample of commercial solder paste and a thickness of 800 microns: (**a**) Step shear rate $\dot{\gamma }$ = 0.5 s^−1^ applied to rejuvenate the sample after loading in the rheometer; (**b**) Time sweep where a SAOS was applied with an amplitude of 0.5% at a frequency of 1 Hz to record the storage (G’, circles) and loss (G’’, squares) moduli during the structural recovery of the sample after the step shear rate; (**c**) Frequency sweep performed with a strain amplitude of 0.5% to measure the linear viscoelastic properties of the sample; (**d**) logarithmic stress ramp performed to measure the yielding and flow behavior of the sample. Finally, a time sweep was performed, as in (**b**), right after the stress ramp (see inset in (**d**)) in view to measure the structural recovery of the paste after flow cessation.

Rheological testing of ECA should mimic the shear rates and stresses applied during SMT and quantified above. In addition to the determination of the yield stress and flow behavior, the rheological characterization should also ideally give access to the recovery time of the material after flow cessation and to its characteristic response time upon imposition of flow or stress. The values of the SMT rheological parameters computed from the analysis presented above are gathered in Table 2. Note that the magnitudes of the shear rates and stresses displayed in Table 2 for the printing are in harmony with the values computed from the modelling of shear rates and stresses taking place within 1 mm close to the tip of the squeegee [9,12,17]. The rheological protocol is presented in Figure 2 together with the rheological response of the commercial solder paste, whose rheology is to be benchmarked.

The protocol started with the application of a step shear rate $\dot{\gamma }$ = 0.5 s^−1^ during 120 s (see Figure 2a), followed by a small amplitude oscillatory shear (SAOS) test where both strain amplitude and frequency were maintained at 0.5% and 1 Hz, respectively, and a data point was measured each 7 s during 5 min (see Figure 2b). The storage modulus $G_{\prime }=\frac{\sigma_{0}}{\gamma_{0}}\cos\delta$ and the loss modulus $G_{\prime \prime }=\frac{\sigma_{0}}{\gamma_{0}}\sin\delta$ were computed from the ratio of the measured oscillatory shear stress amplitude *σ*_0_ over the applied 0.5% SAOS strain *γ*_0_, taking into account the phase shift *δ* between the oscillatory shear stress response and the imposed SAOS strain. The purpose of applying a step shear rate is to rejuvenate the paste and erase all mechanical and structural history associated with the sample loading. The value of $\dot{\gamma }$ = 0.5 s^−1^ was chosen as this shear rate is small enough to avoid any fracture or shear instabilities, as established in preliminary trials, while the shear-induced rejuvenation enhances the experimental reproducibility in the rheological characterization of colloidal suspensions [16,18]. The duration (120 s) of the step shear rate was much larger than the estimate of the rest time *t_r_* after dispensing. However, such long shearing time was needed to ensure the reading of a steady-state shear viscosity, which indicates the establishment of a constant and reproducible shear-induced structure before subsequent rheological testing. Data in Figure 2b indicate that the structural build-up of the paste following the rejuvenation reached an apparent equilibrium within the 5 min of testing, which thus allowed the start of the rheological characterization of the samples.

A SAOS frequency sweep was thus performed from 100 Hz down to 0.1 Hz to record the mechanical spectra of the samples (see Figure 2c), where *G’* and *G’’* was computed as above for each applied frequency. Qualitatively similar spectra were measured for all samples and were reminiscent of solid-like viscoelastic materials. The value *G*_0.1_ of the storage modulus measured at 0.1 Hz can be used to quantitatively compare the elasticities of samples. Then a ramp in shear stress from 100 Pa to 2500 Pa was performed, with 20 stress values per decade. Each stress was applied during 10 s, whereas a shear viscosity was computed from the average of the shear rate data measured during the last second of applied stress. This test allows for the experimental determination of the dynamic yield stress *σ_y_* (also called shearing strength), which shows up as a stress plateau in the low shear rate regime (see Figure 2d), while minimizing the occurrence of shear localization [19]. The test also returns a shear viscosity *η_x_* for a range of measured shear rates *x,* which match those encountered during SMT. 10 seconds stress loading was chosen, as the equipment can apply a steady controlled stress within a much shorter time, whereas a sufficient angular deflection or rotation can be detected by the optical encoder of the stress rheometer at smaller stresses. However, measurements within times as short as tens of milliseconds could not be performed to mimic the characteristic times of the stencil printing. Finally, the test performed in Figure 2b was reproduced to measure the recovery of the sample after flow cessation in the stress ramp (see inset to Figure 2d). The kinetics of such recovery, *t_r_*, can be compared with the characteristic times *t_l_*_1_ and *t_l_*_2_.

To summarize this section, the protocol presented in Figure 2 was adopted to rejuvenate samples and, thus, improved experimental reproducibility, while it opened the route to the determination of various viscoelastic properties under rheological conditions matching the rheology of SMT. These properties, namely, *G*_0.1_, *σ_y_*, *η_x,_* and *t_r_*, are known to be key rheological functions characterizing yield stress materials used in printing application [19].

### 3.3. Rheological Results and Analysis

**Figure 3:**
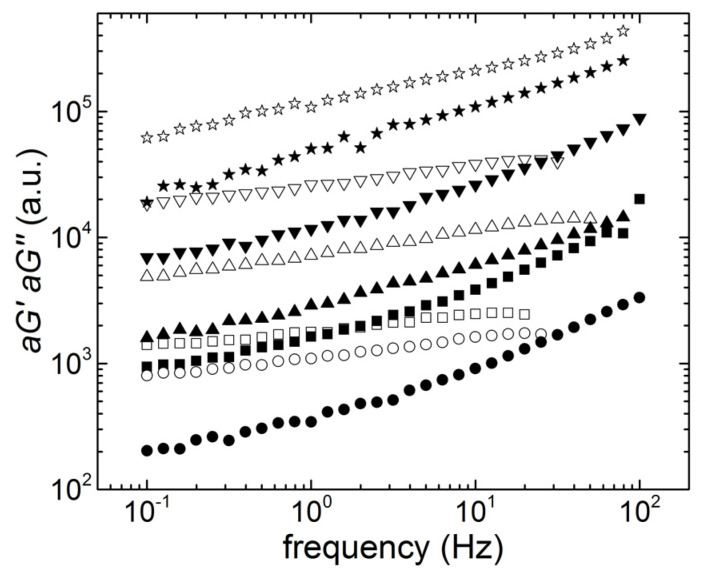
Mechanical spectra (G’: empty symbols, G’’: solid symbols, a.u.: arbitrary units) of the commercial solder paste (squares, shift factor *a* = 0.75), of G2SW02 (circles, shift factor *a* = 0.85), of G2SW05 (up triangles, shift factor *a* = 1.5), of G5SW02 (down triangles, shift factor *a* = 8.5), and of G5SW05 (stars, shift factor *a* = 11) measured with a sample thickness of 800 microns.

Figure 3 presents the mechanical spectra of the materials listed in Table 1. The curves were vertically shifted by a factor *a* to clarify the plot and offer a qualitative comparison of the spectra without overlapping of data points. These were all reminiscent of viscoelastic solids where the storage modulus *G’* was larger than the loss modulus *G’’* at lower frequencies, but both depended on the frequency.

The commercial solder paste showed a crossover between G’ and G’’ at a lower frequency *f_c_* than the crossover frequencies measured with all ECA. This indicated a solid behavior, which extended from long times up to relaxation times of 1 s (*f_c_* = 1 Hz for the commercial paste), in contrast to the ECA, which behaved as solids on a wider spectrum of relaxation processes, up to relaxation times of the order of 0.05 s. Note that no crossover was measured for the ECA that had the largest content in carbon conductive fillers, which suggested that this material was solid-like up to a characteristic relaxation time of the order of 10 milliseconds.

**Figure 4:**
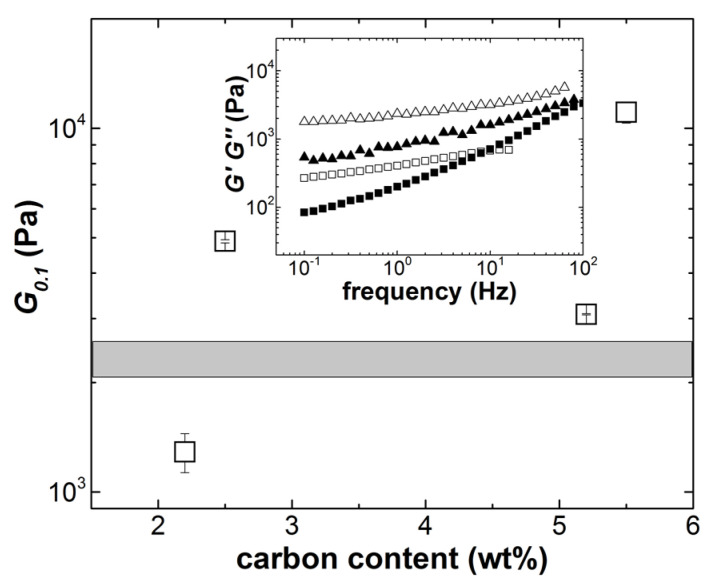
Mean elastic shear modulus *G*_0.1_ (measured at 0.1 Hz with a sinusoidal shear strain amplitude of 0.5%) as a function of the total content in GNP and SWCNT in ECA. Error bars were computed from experiments carried out at two different sample thicknesses. The grayed area represents the range of *G*_0.1_ measured for the commercial solder paste. Inset: mechanical spectra of 0.2 wt.% (squares) and 0.5 wt.% (triangles) SWCNT suspended in epoxy and measured with a sinusoidal shear strain amplitude of 0.5%.

Values of *G*_0.1_ are listed in Table 3, whereas Figure 4 shows its dependence with the total content of carbon nanoparticles. Errors reported for *G*_0.1_ were computed from tests performed at 800 and 300 microns. They represented less than 10% of the value of the shear modulus, which is the accepted error range for rotational rheometry [20].

The solid character in ECA builts on the elastic network of percolated SWCNT existing at SWCNT content as low as 0.2 wt.%. This is illustrated in the inset to Figure 4, which shows the viscoelastic solid-like mechanical spectra of SWCNT suspended in epoxy. In addition, *f_c_* in G2SW02 and G5SW02 were related to the SWCNT content, as their values were nearly similar to the crossover frequency showing up in the mechanical spectrum of the 0.2 wt.% SWCNT suspended in epoxy (see inset to Figure 4). Essentially, the structural information conveyed by the mechanical spectra displayed in Figure 3 and Figure 4 was that the elastic network structure related to the SWCNT content. The elasticity of the hybrid network of GNP and SWCNT was not proportional to the total content of conductive carbon fillers (see Figure 4). This result, together with the SWCNT network structure inferred from the mechanical spectra, suggested that the GNP reinforced the SWCNT network.

Coming back to the rheological benchmarking of ECA with the commercial solder paste, only the ECA formulated with the smallest amount of carbon nanoparticles showed a smaller *G*_0.1_. This does not mean that this formulation was not stencil printable, as the literature does not detail the relationship between the paste elastic shear modulus and its printability [4,5,13,19]. However, as much as the SMT process was concerned, and assuming that all samples did not yield under the weight of electronic components, the lower *G*_0.1_ of sample G2SW02 implied that the printed ECA deposit will deform more than the benchmark deposit during the conveying of the PCB from the pick-and-place machine to the reflow soldering oven.

**Figure 5:**
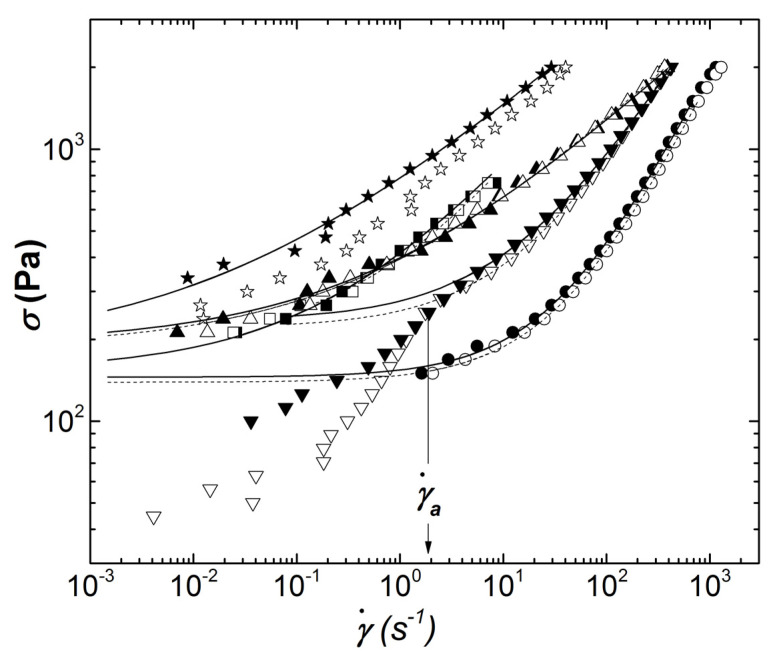
Flow curves measured during stress ramps performed with a gap of 800 microns (solid symbols) and a gap of 300 microns (empty symbols) for SAC305 (squares), G2SW02 (circles), G2SW05 (up triangles), G5SW02 (down triangles), and G5SW05 (stars). Full and dotted lines are fits of Equation (1) to the corresponding data, solid and empty symbols, respectively.

The flow properties measured through the ramp in shear stresses are depicted in Figure 5.

The flow curves exhibit a stress plateau at smaller shear rates before a monotonic increase in stress with larger shear rates. This behavior indicated a solid-to-liquid transition, which is characteristic of yield stress fluids. Sample G5SW02 showed a peculiar behavior, which can be described in three flow regimes. At large stresses, the flow curves measured with different sample thicknesses coincided and showed an onset for plateauing at smaller stress before exhibiting a kink. The kink showed up when a critical shear rate $\dot{\gamma_{a}}$ was reached (see the vertical arrow in Figure 5). At stresses below the kink, a second yield stress behavior was characterized, which strongly depended on the sample thickness. This thickness dependence and the three flow regimes are the hallmark of slip at the shearing wall–sample interface, which has been reported for various types of highly filled suspensions [21]. The yield stress *σ_y_* of sample G5SW02 can, however, be computed by fitting the slip-free regime (for shear rates in excess of $\dot{\gamma_{a}}$) to the Herschel–Bulkley equation, which reads:

(1) $$\sigma =\sigma_{y}+K{\dot{\gamma }}^{n}$$

where *K* is the flow consistency and *n* the shear thinning exponent. Fits of Equation (1) to the high-stress regime of G5SW02 data are presented in Figure 5, together with the fits to other flow curves. The computed yield stresses *σ_y_* are reported in Table 3 with the error bars resulting from the quality of the fit. Note that the latter are as big as the standard error computed from the two tests performed at different sample thicknesses, which validated the bulk flow of material in the high-stress regime.

Sample G5SW05 showed a thickness-dependent, non-linear rheology, which again suggested that slip occurred below and above yielding. This was expected, as slip velocity was found to depend linearly on the suspension shear modulus [22]. Thus, the more elastic ECA formulated with more SWCNT slipped more than sample G5SW02. However, a slipping yield stress was computed from the fit of Equation (1) to the data measured with a sample thickness of 800 microns.

Whereas shear rates as large as 1000 s^−1^ could be measured with the ECA formulated with the lower content in carbon nanoparticles, shear fracture did impede the proper rheological characterization of the remaining samples at shear rates in excess of 400 s^−1^ (see Figure 5). Therefore, the fits of Equation (1) to the data displayed in Figure 5 were used to compute the shear viscosities $\eta_{x}=\sigma \left(x\right)/x$ at shear rates *x* relevant to the SMT process. The computed *η_x_* are listed in Table 3 with the corresponding errors estimated as for *σ_y_*. Overall, data in Table 3 confirmed the rheological message conveyed by Figure 5: The ECA formulated with 2 wt.% GNP and 0.5 wt.% SWCNT showed the flow behavior that best matched the commercial solder paste rheology at lower shear rates. The yield stresses reported in Table 3 are smaller than the stresses involved during the placement of electronic components. This implies the flow of all materials listed in Table 3 during this step of the SMT process. ECA made of more than 5 wt.% carbon nanofillers exhibited high shear viscosities, which matched the high shear viscosity of the commercial solder paste. As such, ECA with more than 5 wt.% carbon could also be good candidates for SMT, in particular if one considers that wall slip will compensate the large viscosity measured at shear rates corresponding to the stencil–PCB separation. A dedicated study of slip effects at gaps relevant for this part of the process could help clarify the applicability of G5SW02 and G5SW05 to the SMT.

**Figure 6:**
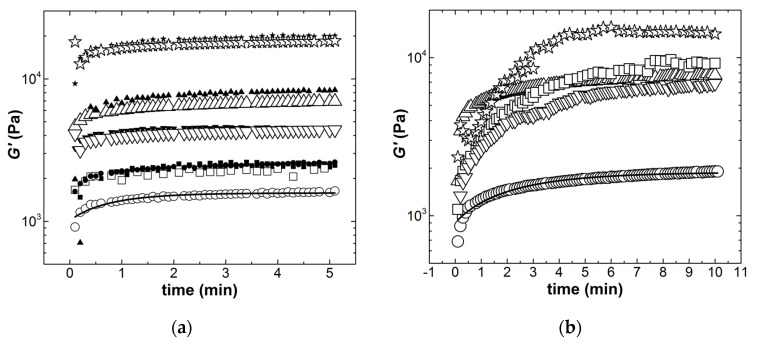
Structural recovery (time evolution of the shear modulus *G’* measured with a sinusoidal strain amplitude of 0.5% and a frequency of 1 Hz): after the application of a step shear rate $\dot{\gamma }$ = 0.5 s^−1^ (**a**); after imposing the largest stress during the stress ramps reported in Figure 6 (**b**). Empty and filled symbols correspond to tests reproduced with different thicknesses for the commercial solder paste (squares), G2SW02 (circles), G2SW05 (up triangles), G5SW02 (down triangles), and G5SW05 (stars). The lines are exponential fits to the data recorded with G2SW02.

We are now left with the quantification of the recovery of the materials after flow cessation. Figure 6 presents the results from the recovery experiments performed after the pre-shear of 0.5 s^−1^ and after the largest stress imposed during the ramp in stress.

The time evolution of the storage modulus *G’* is only reported in Figure 6 as all samples showed *G’* > *G’’* already for the first data point collected at 10 s after flow cessation. This indicates that all ECA recovered a solid-like behavior within 10 s, which matched the time scale of the commercial solder paste. Results from tests carried out at different sample thicknesses are also reported in Figure 6a to show the satisfactory reproducibility of the recovery data. This confirms that the first step of the rheological protocol was efficient in erasing any flow history induced by the loading of samples in the rheometer. The recovery of the samples was fitted with an exponential growth function, and the resulting relaxation times *t_r_* are reported in Table 3. Note here that the errors reported for *t_r_* measured at lower shear rates were computed from the statistical analysis of the duplicated tests reported in Figure 6a. These errors were of the same magnitude as the fitting errors (compared with those reported for *t_r_* computed at larger shear rates), which again underlines the satisfactory reproducibility of relaxation data after imposing a small shear rate of 0.5 s^−1^. Overall, all ECA showed a recovery time at lower shear rates, which matched *t_r_* measured for the commercial solder paste. The relaxation times measured at larger shear rates were significantly larger. This was expected because, in highly filled colloidal suspensions, the recovery is known to strongly depend on the flow-induced structure achieved prior to flow cessation [16], as it connects to the thixotropic restructuring time [19]. The longer restructuring relates to the larger structural break-up achieved under flow. The values reported for *t_r_* in the last column of Table 3 for the ECA were of the same magnitude as for the commercial paste, whereas the latter was submitted to smaller shear rates during the ramping of stresses (see Figure 5). Therefore, all formulated ECA should comply with the structural recovery necessary after stencil printing and before the placement of electronic components. Note also that all times reported in Table 3 are faster than the processing times *t*_l1_ and *t*_l2_, which confirms the ECAs’ compliance with the process in terms of structural recovery.

Table 3 summarizes the rheological analysis performed here. Sample G2SW02 showed the worst rheological benchmarking with the commercial solder. Sample G5SW05, though showing a significantly larger elastic modulus, showed flow properties (yield stress and shear thinning) that were closely matching the flow behavior of the commercial paste. However, the underlying slip phenomena need to be further assessed before concluding on the ability of G5SW05 to be processed with the present SMT. The two remaining ECA exhibited a rheology that either nicely matched the commercial paste rheology (G2SW05) or was adequate to the rheology of the SMT process.

### 3.4. Electrical and Thermal Characterizations

Electrical characterization was performed for all hybrid formulations and additionally for two composites without GNP but with the same composition of SWCNT, 0.2 and 0.5 wt.%. Electrical resistivity and electrical conductivity results are presented in Table 4. The electrical conductivity of the composites with both SWCNT concentrations presented an increase in the electrical conductivity upon addition of GNP. While the composites with 0.2 wt.% SWCNT showed a marginal increase in electrical conductivity that increased with GNP composition, the composite with 0.5 wt.% SWCNT presented a larger increase of the electrical conductivity when GNP was added, however, remaining almost invariant with GNP amount.

**Figure 7:**
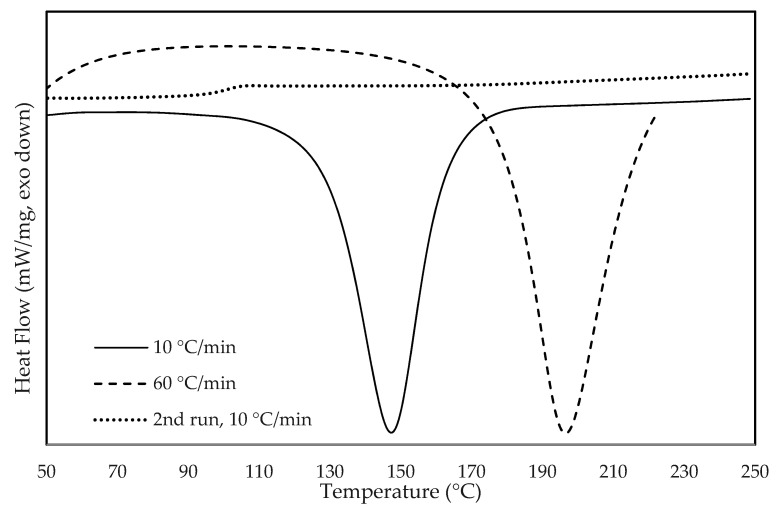
Heat flow curves of sample G2SW05 at the scan rate of 10 °C.min^−1^ (full line) and of 60 °C.min^−1^ (dashed line), and for the second run (dotted line), at 10 °C.min^−1^, after curing during the scan at 60 °C.min^−1^.

Figure 7 presents the DSC curves of the composite G2SW05 obtained at the heating rates of 10 °C.min^−1^ (to measure the cure enthalpy) and 60 °C.min^−1^ (designed to reproduce the thermal profile of the reflow oven). Both curves showed an exothermic peak that occurred around 150 °C for the scan at 10 °C.min^−1^, and 190 °C for the scan at 60 °C.min^−1^. The second scan at 10 °C.min^−1^, carried out on the sample that was first heated at 60 °C.min^−1^, showed that the composite was fully cured under the conditions of the first heating step. This curve depicted the glass transition (≈100 °C) of the ECA cured at a heating rate of 60 °C.min^−1^.

Table 5 and Table 6 present the results of the thermal characterization of all the ECA prepared and the neat epoxy resin. The results obtained for the cure analysis are shown in Table 5, reporting the thermal characteristics of the cure measured on the DSC scans performed at 10 °C.min^−1^. The curves obtained were similar for all the composites. Table 5 shows that the peak temperature and the enthalpy of the cure reaction were not significantly changed by the presence of the carbon nanomaterials while the onset temperature was slightly shifted upwards by approximately 6%.

The results obtained for the scans at 60 °C.min^−1^, presented in Table 6, also depicted a similar peak temperature for the composites and neat resin and an upshift of the composites’ onset temperature. Isothermal tests at 150 °C presented a cure peak at 2.1 min for the neat resin and 2.3 to 2.4 min for the ECA.

## 4. Conclusions

The rheological analysis of a SMT process used in a specific factory plant was performed. Specific ranges of shear rates, stresses, and processing times were computed and then used to design a rheometrical protocol. The latter was employed to assess the rheological properties of several ECA and of a commercial solder paste used for SMT. For each material, the elastic shear modulus *G*_0.1_, the yield stress, the shear viscosities at selected shear rates, and the time needed for structural recovery after steady shear were measured. This rheological study showed that the ECA prepared with 0.5 wt.% SWCNT and 2 wt.% GNP was the best rheological match to the commercial solder paste. Adding larger amounts of GNP can lead to slip issues associated with more elastic pastes.

The electrical characterization revealed that the prepared adhesives are conductive, and the conductivity improves with increase of SWCNT amount. An increase of electrical conductivity was observed with the addition of GNP to the composite.

The cure behavior of the resin was not impacted by the presence of the carbon nano fillers, and the formulated resins are fully cured when subjected in the DSC to a temperature profile program similar to the reflow oven temperature profile used in the factory plant.

The main output of this study is that an ECA (sample G2SW05) was formulated for SMT application. This ECA shows optimal electrical conductivity and rheological properties matching those of a commercial solder paste and is the best candidate for testing in a SMT equipment.

## Figures and Tables

**Table 1:** Samples’ acronyms and formulation.

Sample	GNP (wt.%)	SWCNT (wt.%)
SAC305	0	0
G2SW02	2	0.2
G2SW05	2	0.5
G5SW02	5	0.2
G5SW05	5	0.5

**Table 2:** Rheological analysis of the SMT with the quantification of the characteristic times of the process steps during which the tabulated shear rates and stresses are applied.

Process Steps	Shear Rates (s^−1^)		Shear Stresses (MPa)		Characteristic Time (s)	
	Min	Max	Min	Max	Min	Max
printing	312	1000	-	-	0.012	0.304
Time lapse *t_l_*_1_	0	0	0	0	1	30
separation	0.16	16	-	-	0.01	0.08
Time lapse *t_l_*_2_	0	0	0	0	seconds	minutes
placement	-	-	0.05	62.5	0.1	1

**Table 3:** Rheological properties of ECAs and commercial solder paste SAC305.

Samples	*G*_0.1_ (Pa)	*σ_y_* (Pa)	*η*_0.16_ (Pa.s)	*η*_16_ (Pa.s)	*η*_312_ (Pa.s)	*η*_1000_ (Pa.s)	*t_r_* (s)	
							$\dot{\gamma }$ = *0.5 s*^−1^	*high*
SAC305	2363 ± 232	174 ± 15	1633 ± 111	61 ± 6	11.3 ± 2.3	6.1 ± 1.5	0.81 ± 0.05	4.18 ± 0.20
G2SW02	1288 ± 159	142 ± 6	901 ± 27	13.4 ± 0.5	2.77 ± 0.14	1.91 ± 0.1	0.84 ± 0.10	2.66 ± 0.12
G2SW05	4889 ± 52	190 ± 9	1855 ± 24	46.89 ± 0.14	5.84 ± 0.01	2.67 ± 0.01	0.81 ± 0.18	2.11 ± 0.14
G5SW02	3079 ± 12	227 ± 7	1493 ± 77	28.2 ± 1.7	5.54 ± 0.02	3.4 ± 0.1	0.79 ± 0.14	3.58 ± 0.13
G5SW05	11076 ± 741	186 ± 26 ^1^	3202 ± 322 ^1^	105 ± 13 ^1^	13.4 ± 2.06 ^1^	6.06 ± 1.02 ^1^	0.79 ± 0.07	3.09 ± 0.29

^1^ Computed from the fit of Equation (1) to the flow curve measured at 800 microns.

**Table 4:** Electrical properties of the ECAs prepared.

Sample	Electrical Resistivity (Ω.m)	Electrical Conductivity (S.m^−1^)
G0SW02	3.48 ± 1.00	0.302 ± 0.067
G0SW05	1.21 ± 0.39	0.896 ± 0.287
G2SW02	3.15 ± 0.43	0.321 ± 0.041
G2SW05	0.75 ± 0.24	1.427 ± 0.409
G5SW02	2.32 ± 1.03	0.507 ± 0.238
G5SW05	0.77 ± 0.20	1.375 ± 0.421

**Table 5:** Thermal characteristics of the composites from the DSC scan at 10 °C.min^−1^.

Sample	Peak Temperature (°C)	Enthalpy (J.g^−1^)	Onset Temperature (°C)
Neat resin	147.0 ± 0.2	−302.7 ± 13.4	121.1 ± 0.4
G2SW02	149.3 ± 0.5	−293.3 ± 24.6	130.4 ± 1.7
G2SW05	147.7 ± 0.6	−293.6 ± 8.4	128.3 ± 1.1
G5SW02 *	149.4 ± 0.4	−300.9 ± 19.5	129.3 ± 0.6
G5SW05	146.4 ± 0.1	−292.1 ± 27.5	125.3 ± 0.7

* This sample was tested after storage at −18 °C for 3 months.

**Table 6:** Thermal characteristics of the composites from the DSC scan at 60 °C.min^−1^ (peak and onset temperature) and the isothermal test at 150 °C (peak time).

Sample	Peak Temperature (°C)	Onset Temperature (°C)	Peak Time (min)
Neat resin	184.8 ± 0.4	160.5 ± 1.4	2.1
G2SW02	190.03 ± 0.9	172.2 ± 1.5	2.4
G2SW05	188.8 ± 1.4	172.2 ± 2.5	2.3
G5SW02 *	193.6	174.9	2.4
G5SW05	186.9 ± 0.7	164.8 ± 1.4	2.3

* This sample was tested after storage at −18 °C for 3 months.
